# Anthelmintic resistance status of goat gastrointestinal nematodes in Sing Buri Province, Thailand

**DOI:** 10.14202/vetworld.2022.83-90

**Published:** 2022-01-20

**Authors:** Niorn Ratanapob, Nattanan Thuamsuwan, Suporn Thongyuan

**Affiliations:** 1 Department of Large Animal and Wildlife Clinical Sciences, Faculty of Veterinary Medicine, Kasetsart University, Malaiman Road, Kamphaeng Saen, Nakhon Pathom, Thailand; 2 Sing Buri Provincial Livestock Office, Department of Livestock Development, Sing Buri-Suphan Buri Road, Ton Pho, Mueang district, Sing Buri, Thailand; 3 Department of Veterinary Public Health, Faculty of Veterinary Medicine, Kasetsart University, Malaiman Road, Kamphaeng Saen, Nakhon Pathom, Thailand.

**Keywords:** anthelmintic resistance, fecal egg count, gastrointestinal nematode, goat

## Abstract

**Background and Aim:**

Promotions of goat farming by both public and private sectors encouraged considerable goat raising in central Thailand. Gastrointestinal nematodes (GIN) infection is a major health and economic problem; however, evidence of resistance to broad-spectrum anthelmintics is frequently reported. Investigation of anthelmintic resistance (AR) status and identification of factors related to the development of AR is important components for sustainable GIN control. However, no information is available on this topic in the study area. The present study aimed to gather information on GIN control practices and to evaluate the effectiveness of albendazole, ivermectin, and levamisole for treating GIN infestation in goat herds in Sing Buri Province.

**Materials and Methods:**

Twenty-nine herds were randomly selected. Information on management practices was collected by face-to-face interview using a structured questionnaire. Three field experiments for routinely used anthelmintics, including albendazole, ivermectin, and levamisole were conducted from June 2019 to November 2019. Fecal samples were collected pre- and post-treatment and examined for fecal egg count reduction to determine the status of anthelmintic resistance of goat GIN.

**Results:**

Several improper practices were identified that lead to AR, especially chronic use of albendazole and ivermectin. All herds were considered resistant to albendazole and ivermectin, and levamisole resistant nematodes were detected in two herds. AR was strongly linked with the continuous use of anthelmintics.

**Conclusion:**

Levamisole, which was still effective in the province, should be used with caution to minimize the selection of resistant strains. Farmers should be provided with updated information for sustainable parasite control. Further, the efficacy of anthelmintics should be routinely monitored.

## Introduction

Gastrointestinal nematodes (GIN) are a major health issue for goats. GIN infestation causes significant economic loss and welfare concerns worldwide. Control of GINs requires at least three elements: (1) Eliminating GINs from hosts, (2) avoiding exposure of hosts to an infective stage of GINs, and (3) enhancing host resistance [1]. However, most farmers rely mainly on the use of anthelmintics [2], which is not sustainable for GIN control. Three major groups of broad-spectrum anthelmintics, namely, benzimidazoles, macrocyclic lactones, and imidazothiazoles are available for GIN control in small ruminants [3]. In Thailand, benzimidazoles and macrocyclic lactones are commonly used in goat herds. Anthelmintic resistance (AR) to all three groups of anthelmintics are described in several goat raising areas globally [2,4-8]. Evidence of resistance to all three groups of anthelmintics is frequently reported in goats in Thailand [9-11]. Various approaches are used to detect AR, including fecal egg count reduction (FECR), egg hatching, larval development, and molecular-based tests [12,13]. The FECR test is limited by high labor-intensity and inter-animal variation, yet it is the recommended method for AR investigations [2]. Improper anthelmintic use, such as underdosing, and continuous and frequent use of a single anthelmintic, is common practices that contribute to AR. Moreover, AR parasites can be dispersed by introducing new animals without sufficient quarantine [4,14] and improper pasture management [4]. Investigation of AR status and identification of factors associated with the development and dispersal are essential components of helminth control [1,15]. The results of this study will be beneficial in managing AR and creating practical, sustainable strategies for GIN control.

Promotions of goat farming by both public and private sectors encouraged considerable goats raising in central Thailand. GIN infection is a prevalent problem in the central region of the country [16,17], but no study on AR is reported for this area. However, based on observation of farmers and local veterinarians, existence of AR in this area has been considered.

The present study aimed to identify management practices for GIN control and evaluate AR status of goat GINs for the three major groups of broad-spectrum anthelmintics; benzimidazoles (albendazole), macrocyclic lactones (ivermectin), and imidazothiazoles (levamisole) in goat herds raised in In Buri district, Sing Buri Province.

## Materials and Methods

### Ethical approval and Informed consent

The study protocol was reviewed and approved by the Animal Care and Use for Scientific Research Committee, Kasetsart University (U1-07635-2561). The procedures used in this study adhere to the tenets of the Declaration of Helsinki. Goat farmers were informed about the purpose of the study and asked to participate in the evaluation of anthelmintics and respond to questions on farm management and practices for GIN control.

### Study period and area

The study was conducted from June to November 2019 in two sub-districts of In Buri district (15° 0̍ 2̎ N, 100° 19̍ 3̎ E), Sing Buri Province, Thailand. This area is a flat river plain in the central part of the country. The average annual temperature is 28-30°C, with minimum and maximum temperatures of 22°C in December and 34°C in April, respectively. The average annual rainfall is 100-1100 mm. Over one-fourth of goat farmers and 36% of goats in Sing Buri Province are located in these two sub-districts, and local goat farmer cooperative associations are present in each area.

### Experimental procedures

Twenty-nine meat goat herds from two sub-districts were randomly recruited from 82 registered herds in the database of the Department of Livestock Development, Thailand, using simple random sampling. With the consent, face-to-face interviews were conducted with farmers on their farms using a structured questionnaire with closed-ended and open-ended questions to collect the information including herd characteristics, management practices, deworming practices, and health problems associated with GIN infection.

Characteristics of participating herds are displayed in Table-1. The primary purpose of goat raising was selling meat to markets. The goats in herds ranged from 16 to 200, with a median of 38. Median goat farming experience was 3 years. Most farmers allowed goats to graze on common pastures, attempting to rotate grazing areas every few days. Grazing was allowed after 8 am in every herd, and all goats returned from grazing before 6 pm.

**Table-1:** Characteristics of 28 goat farms in In Buri district, Sing Buri Province.

Characteristics	Frequency	Percentage
Gender of the owner		
Male	19	67.86
Female	9	32.14
Education of the owner		
Lower than secondary school	21	75.00
Secondary school or higher	7	25.00
Training experience of the owner		
Never	10	35.71
Yes	18	64.29
Goat farming experience of the farmer		
<2 years	14	50.00
2–4 years	3	39.29
>4 years	11	10.71
Farm management practices		
Major purpose		
Meat	27	96.43
Breeder	1	3.57
Breed		
Pure breed	1	3.57
Mixed breed	27	96.43
Introducing new goat in farm		
Male	24	85.71
Female	10	35.71
Grazing		
No grazing	3	10.71
Own grazing area	4	14.29
Grazing on common pastures	21	75.00
Sharing with other goats	13	61.90
Sharing with cattle	4	19.05
Sharing with both goat and cattle	4	19.05
Providing concentrate	24	85.71
Providing forage	22	78.51
Providing mineral supplement	26	92.86

### FECR

Three field experiments were sequentially conducted to evaluate the efficacy of albendazole, ivermectin, and levamisole from June to November 2019. Experiments were spaced at least 8 weeks apart from the latest deworming to ensure that the effects of the previous anthelmintic treatment had subsided [3]. Ten adult goats for each herd were conveniently selected for each experiment, as suggested for performing FECR tests without untreated control [12]. Pre-treatment individual fecal samples were collected directly from the rectum for egg count. Animals were singly treated with a local brand of anthelmintics at the manufacturer’s recommended doses based on estimated body weights as farmer’s routine practices (Table-2). Individual goats were identified by spray markings and ear tags.

**Table-2:** Dose, route of administration, and duration until post-treatment sample collection for the three anthelmintics used in the study.

Anthelmintic	Dose based on the product prescription	Route	Duration (days)
Albendazole	5.625 mg/kg	Oral	8-10
Ivermectin	0.2 mg/kg	Subcutaneous	14-17
Levamisole	5 mg/kg	Subcutaneous	3-7

Only herds with mean pre-treatment fecal GIN egg counts ≥150 eggs/g were considered for post-treatment sample collection [18]. Individual goats with pre-treatment fecal GIN egg counts ≥50 eggs/g were selected for post-treatment samples collection. The duration between pre- and post-treatment sample collection depended on anthelmintic (Table-2) [12]. The number of GIN eggs per gram of feces was determined using the Modified McMaster technique [19]. The minimum detection limit for this technique is 50 eggs/g of feces. FECR at each herd, the parameter of choice for an AR survey [2] was calculated as:

Fecal egg count reduction % = (1/n) Σ(100×(1−[Ti_2_/Ti_1_])

Where, Ti_1_ and Ti_2_ are fecal egg counts, pre- and post-treatment of individual goat, respectively, and n is the number of samples from the herd [20].

### Statistical analysis

Statistical analyses were performed to identify factors associated with multi-AR status. The treatment was considered AR when FECR is <95% and lower end of the 95% confidence interval (CI) is <90%. If one condition is met, AR is suspected [3]. Data from the questionnaire were analyzed with descriptive statistics. A Chi-square test for independence was carried out to test the relationship between herd characteristics, management practices, deworming practices, and health problems related to GIN infection and AR status. Binary logistic regression was used to identify AR status factors and presented with an odds ratio (95% CI). The p values were two-tailed, and p<0.05 was considered statistically significant. Data analysis was performed using Stata version 17.0 (StataCorp LLC, College Station, Texas, USA).

## Results

From all 29 participating farmers, 27, 24, and 23 agreed to participate in the evaluation of the effectiveness of albendazole, ivermectin, and levamisole, respectively. Twenty of 29 farmers participated in all three experiments. Reasons for non-participation included no longer having goats, receiving the last anthelminthic treatment <8 weeks after previous treatment, and unavailable of farmers. Twenty-eight farmers consented to be interviewed.

Deworming practices are shown in Table-3. All participating farmers often bought and administered anthelmintics themselves. In general, anthelmintics were administered to all goats every 1-3 months, with a median of 4.8 times/year or every 2.5 months. One farmer used anthelmintics only when he noticed that goats displayed poor body conditions or hair coats. In most herds, individual goat weights for dose calculation were visually estimated. Ivermectin was used in all herds, and benzimidazole was used in more than 80% of herds. These anthelmintics had been used since herds were established. One-fourth of farmers reported an experience with levamisole obtained from the Department of Livestock Development. Most farmers who reported using ivermectin administered 2-3 ml for adults, which are higher doses than recommended in the prescription of local produce brands available in this area (0.2 mg/kg).

**Table-3:** Deworming practices of 28 goat farms, In Buri district, Sing Buri Province.

Deworming practices	Frequency	Percentage
Frequency of anthelmintics use in farm		
12 times/year	4	14.29
6 times/year	8	28.57
4 times/year	6	21.43
3 times/year	7	25.00
2 times/year	2	7.14
As required	1	3.57
Body weight estimation		
Every goat (visually)	24	85.71
Only the heaviest goat	4	14.29
Administered albendazole		
Yes	25	89.29
Never applied	3	10.71
Administered Ivermectin		
Yes	28	100.00
Administered levamisole		
Yes	7	25.00
Never applied	21	75.00
Administered combination of albendazole and ivermectin		
Yes	3	10.71
Never applied	25	89.29

Conversely, underdosing and overdosing with benzimidazole were uncommon. Combined administration of ivermectin and benzimidazole was used in a few herds. Four farmers reported no improvement in goat health after routine deworming. Most participating farmers considered diarrhea, stunted growth, and weight loss as health issues in their animals.

**Figure-1:**
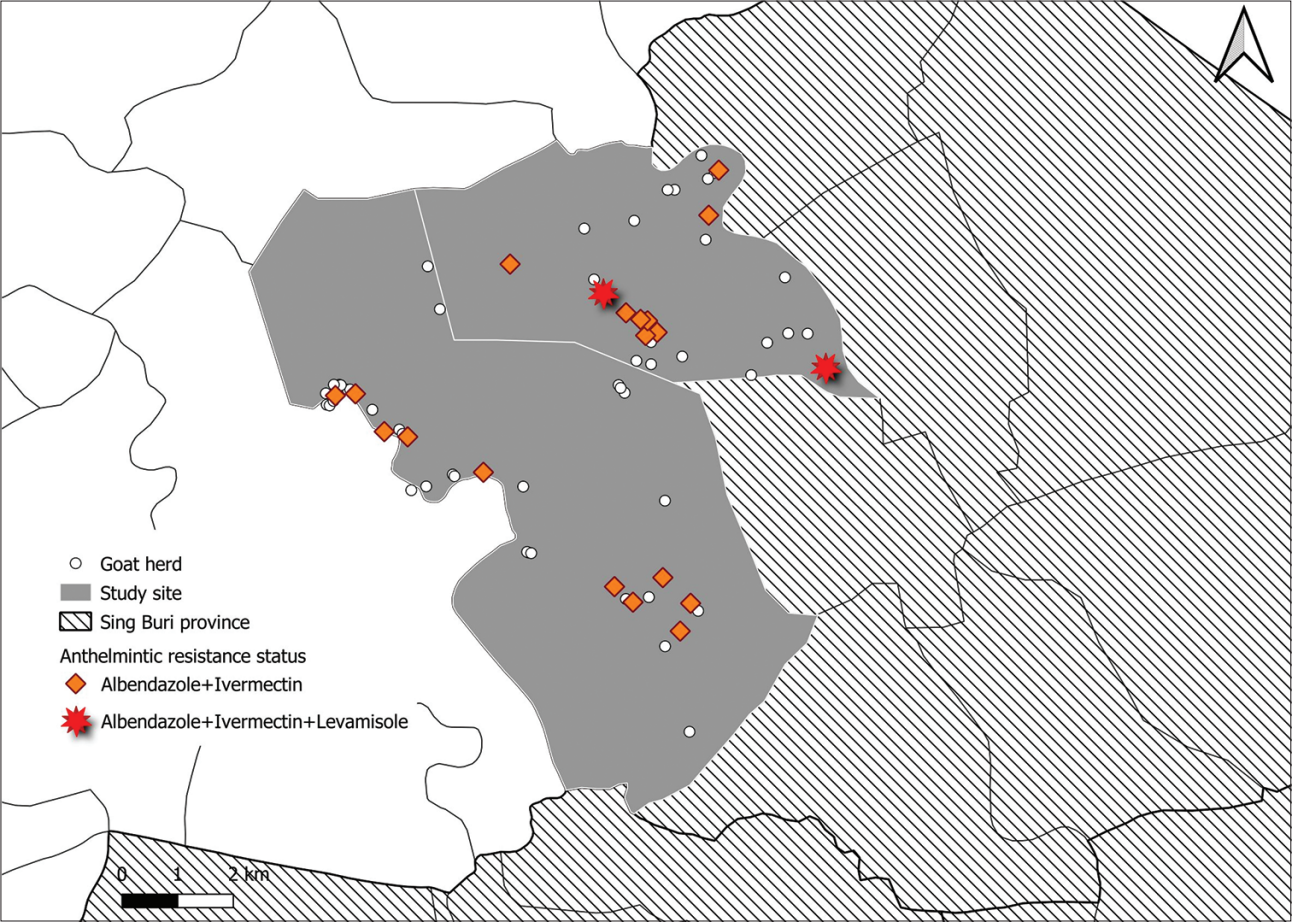
Study area, highlighting locations of 20 herds that participated in all 3 experiments and locations of herds with anthelminthic resistant parasites [Source: The map was constructed using a base map from GADM, version 1.0 for administrative areas (https://www.diva-gis.org/gdata)].

Most eggs detected in fecal samples were GINs, including strongyle-type and *Strongyloides* spp. Eggs of *Moniezia* spp. and oocysts of *Eimeria* spp. were also detected in some samples. Mean pre-treatment fecal GIN egg count was >150 eggs/g of feces in every herd in all three experiments. All herds participating in albendazole and ivermectin treatment showed AR. Medians FECR were −97% and −71%, which reflect increased numbers of eggs post-treatment compared to pre-treatment findings. Only two herds were considered to have levamisole-resistant GINs. The median FECR for levamisole experiment was 97%, with seven herds having 100% mean FECR. Of 20 herds where all three experiments were completed, resistance to albendazole, ivermectin and levamisole was found in two (10%). In addition, resistance to both albendazole and ivermectin was found in the 18 remaining herds (90%) (Table-4 and Figure-1).

**Table-4:** Fecal egg count reduction for albendazole, ivermectin, and levamisole in 29 goat herds in In Buri district, Sing Buri Province.

Herd	Mean				Albendazole (n=27)	Mean				Ivermectin (n=24)	Mean				Levamisole (n=23)
		
	Lower 95% CI	Median	Q1	Q3		Lower 95% CI	Median	Q1	Q3		Lower 95%CI	Median	Q1	Q3	
1	−35.96*	−80.99	−29.67	−33.33	0.00	−238.16*	−654.94	3.26	−92.00	47.37	50.26*	−15.05	100.00	66.66	100.00
2	−241.63*	−744.54	60.56	0.00	70.00	−24.12*	−99.56	16.98	−6.06	42.86	NA	NA	NA	NA	NA
3	−54.90*	−120.91	−40.00	−164.52	7.69	34.76*	−5.56	50.96	28.72	67.06	100.00	100.00	100.00	100.00	100.00
4	−93.58*	−263.89	−15.38	−266.67	69.23	−42.27*	−128.66	14.10	−140.00	50.00	NA	NA	NA	NA	NA
5	−36.14*	−112.66	20.00	−150.00	50.00	−28.91*	−106.23	0.00	−100.00	60.00	99.52	98.57	100.00	100.00	100.00
6	−97.46*	−209.51	−74.72	−185.71	76.19	NA	NA	NA	NA	NA	NA	NA	NA	NA	NA
7	−555.54*	−807.94	−540.00	−644.44	−500.00	−35.52*	−153.50	29.94	−70.00	59.26	99.25	98.50	100.00	97.78	100.00
8	−76.65*	−168.94	−11.90	−185.42	21.93	−182.29*	−318.73	−159.72	−300.00	−30.37	100.00	100.00	100.00	100.00	100.00
9	−475.64*	−807.09	−395.88	−915.38	−166.67	−134.32*	−199.33	−94.23	−244.44	−50.00	99.07	97.25	100.00	100.00	100.00
10	−409.95*	−861.64	−110.71	−180.00	−18.75	−360.86*	−1069.04	10.00	−34.72	42.86	93.86*	88.64	96.30	93.08	100.00
11	−64.92*	−201.99	36.00	−63.64	78.57	−68.62*	−156.87	−41.43	−100.00	25.00	97.50	92.57	100.00	100.00	100.00
12	−260.95*	−580.68	−63.35	−300.00	41.94	−13.94*	−49.74	−2.50	−68.18	31.58	99.54	98.93	100.00	100.00	100.00
13	6.97*	−54.32	−25.00	−60.00	92.00	NA	NA	NA	NA	NA	98.45	95.90	100.00	99.51	100.00
14	−435.10*	−991.92	−77.22	−425.00	16.13	−116.55*	−185.90	−111.11	−169.23	−77.78	99.12	98.19	100.00	98.39	100.00
15	−119.00*	−168.50	−141.66	−150.00	−66.67	−24.16*	−81.22	33.33	−75.00	35.71	99.80	99.40	100.00	100.00	100.00
16	−528.00*	−1016.60	−365.62	−510.26	−119.96	−119.10*	−223.21	−100.00	−204.04	2.64	100.00	100.00	100.00	100.00	100.00
17	−334.23*	−510.89	−366.66	−537.50	−71.43	−59.17*	−161.41	16.67	−162.50	77.78	99.21	97.99	100.00	100.00	100.00
18	−172.74*	−501.79	14.29	−183.33	87.50	−93.10*	−308.62	13.04	−25.00	60.00	100.00	100.00	100.00	100.00	100.00
19	−101.01*	−225.97	−47.37	−233.33	0.00	−192.07*	−−362.89	−107.48	−159.52	−36.67	99.13	97.41	100.00	100.00	100.00
20	−63.94*	−132.02	−100.82	−150.00	44.23	NA	NA	NA	NA	NA	NA	NA	NA	NA	NA
21	−119.41*	−232.54	−50.90	−200.00	14.29	NA	NA	NA	NA	NA	100.00	100.00	100.00	100.00	100.00
22	−21.96*	−74.79	−−19.84	−93.34	44.28	−72.86*	−167.33	0.00	−215.38	45.83	100.00	100.00	100.00	100.00	100.00
23	−96.40*	−171.49	−74.16	−119.05	−28.57	−116.13*	−157.31	−100.00	−131.58	−93.33	96.60	91.15	100.00	100.00	100.00
24	−1.47*	−89.10	72.80	−100.00	100.00	23.46*	−23.75	30.65	0.00	33.33	NA	NA	NA	NA	NA
25	−71.03*	−160.06	−142.86	−160.00	41.18	−211.24*	−398.52	−114.28	−280.68	−50.00	99.28	97.88	100.00	100.00	100.00
26	−128.33*	−365.40	−7.50	−180.00	25.00	−340.00*	−638.86	−158.33	−550.00	−25.00	98.94	97.52	100.00	100.00	100.00
27	−138.31*	−479.22	31.25	−29.41	65.63	−14.38*	−107.90	30.52	−36.66	64.35	99.92	99.77	100.00	100.00	100.00
28	NA	NA	NA	NA	NA	−43.73*	−137.42	37.10	−79.49	53.85	NA	NA	NA	NA	NA
29	NA	NA	NA	NA	NA	NA	NA	NA	NA	NA	100.00	100.00	100.00	100.00	100.00
All	−166.96	−234.38	−101.01	−260.95	−63.94	−103.05	−147.34	−70.74	−158.31	−26.53	96.93	92.49	99.28	98.94	100.00

Q1=First quartile, Q3=Third quartile; NA=Not applicable; * Herd interpreted as having resistance to GIN. CI=Confidence interval

The present study could not statistically identify the contributing factors of AR against albendazole and ivermectin because all participating herds showed resistant GINs to these two anthelmintics. For levamisole, only two herds displayed resistant GINs and they reported applying an anthelmintic once a month. Similar to the other factors, the use of levamisole in the herd was not related to its resistance status (p=0.311). One of these two herds had some experience using levamisole, while another herd had never used it. However, the latter reported introducing new goats into the herd.

Our results demonstrated trends of association between introducing of new goat into the herd, herd size ≥54 animals, number of adult goats ≥38, frequently used of anthelmintic and history of pale mucous membrane and infertile goats within herd and multi-AR status; however, no statistically significant association was presented (Table-5).

**Table-5:** Univariate logistic regression analysis of multi−AR determinants.

Variables	OR	95% CI	p-value
Introducing new goat			
No	1		
Yes	0.857	0.068-20.53	0.907
Size of the herd			
<54 animals	1		
≥54 animals	1.08	0.09-25.61	0.955
Number of juvenile goats in the herd			
<16	1		
≥16	0.26	0.01-3.29	0.317
Number of adult goats in the herd			
<38	1		
≥38	1.33	0.11-31.56	0.826
Grazing with other animals			
No	1		
Yes	0.75	0.03-9.17	0.996
Frequent use of anthelmintics			
No	1		
Yes	0.67	0.05-16.15	0.826
Recently presented of pale mucous membrane goat in the herd			
No	1		
Yes	1.08	0.09-25.61	0.955
Recently presented infertile goat in the herd			
No	1		
Yes	12.00	2.36-218.63	0.401

OR=Odds ratio, CI=Confidence interval

## Discussion

Most farmers accepted that GIN infection was common in their herds. Several approaches were used to address this problem, such as using anthelmintics, alternating classes of anthelmintics, rotational grazing of common pastures, and avoiding grazing in mornings and evenings to minimize parasite exposure. However, the prevalence of GIN infection was still high. Some farmers thought that AR parasites existed in their herds, based on an absence of clinical improvement of goats after treatment, even after increased doses of anthelmintics above doses recommended in prescriptions, especially for ivermectin.

In the present study, we focused on the efficacy of anthelmintics against GINs because all three anthelmintics used are typically effective against this group of parasites. In albendazole and ivermectin experiments, FECRs in almost all herds were negative, indicating that no reduction in their egg excretion after treatment and these anthelmintics might be less efficacious. A similar finding was reported from a study of herds where anthelmintics had been used for long periods. The underlying mechanism of this phenomenon is not understood, and reconsideration of anthelmintic use as a cause is needed [5].

Our findings of AR in GINs concurs with the previous studies on Malaysian and southern Thai goats that reported the lowest degree of resistance for levamisole compared to albendazole and ivermectin [11,21]. An explanation is that levamisole is the least frequently used anthelmintic in participating herds, as revealed from the interview. Albendazole and ivermectin are widely available in the country, including the study area and have been extensively used for many years. Widespread resistance to two anthelmintics is not surprising. In contrast, levamisole was not available in animal supply stores in the study area when the study was conducted. Some farmers infrequently received levamisole from the Department of Livestock Development. Lower resistance to benzimidazoles and macrocyclic lactones than levamisole was reported in a US study, in which levamisole was most frequently used [7].

Frequent anthelmintic use was reported as the most important cause of AR development [18,22,23]. An optimum frequency for dairy goats of 2-3 times a year was proposed by Lespine *et al*. [24]. Anthelmintics were used up to 12 times/year in some small ruminant herds in Malaysia [25]. In the present study, 14.3% of goat herds were administered an anthelminthic every month, including 2 farms which resistance to levamisole was detected. Alternating a few anthelmintic drugs annually could slow AR development [24]. In the present study, an alternating between albendazole and ivermectin was used in most herds. Alteration patterns could not be identified by most farmers. In any case, alternating anthelmintics did not seem helpful in these herds, which might be due to implementing the strategy too late when AR already existed. The irregular alternating might be another explanation for the failure of the strategy.

Underdosing is a major factor in AR development [4,22]; however, we found that it was uncommon in the study population. Most farmers reported overdosing of ivermectin. These farmers may have confronted treatment failure decided on their own to increase doses hoping it would enhance the effectiveness of treatment. Overdosing by farmers might be a reason why the dose of ivermectin used in the present study was not effective in reducing fecal egg count. Doses in prescriptions of available anthelmintics for goats in Thailand are based on doses recommended for sheep, and these doses are lower than suitable doses for goats. Metabolism of anthelmintics in goats is faster than in sheep, and 2 times the recommended dose for sheep should be used in goats [26]. Regarding the double dose recommendation, 36.0% of farmers in this study were considered overdosing their goats with ivermectin. Remarkably, the study in dairy goat farms in Slovakia revealed that using double doses of albendazole (10 mg/kg body weight) for treating goats may underestimate the actual occurrence of low levels of resistant parasites in the population [2].

Typically, farmers select and administer anthelmintics to their goats without supervision by veterinarians. Misuse of drugs, such as inaccurate calculation of doses or improper administration methods, can result in underdosing. Further, farmers mostly purchased anthelmintics from local animal feed and drug stores. Products available in these stores are rarely inspected. Farmers are more likely to buy veterinary products at lower prices to reduce production costs. Low priced anthelmintics might be expired or low quality and promote AR [27]. Differences in efficacy between the brands of anthelmintics were most likely due to variations in quality rather than the administered doses [28], status of the efficacy of the anthelmintics widely used in the local markets should be monitored. In addition, providing appropriate healthcare supervision to farmers would be beneficial to slow AR development that results from erroneous practices [4].

An influence of pasture rotation on delaying AR is well recognized [4,22,29]. Unfortunately, herds in the present study cannot fully implement such a system because of restricted grazing areas. Common pastures are used extensively because most farmers do not possess any land or cannot manage lands affected by drought.

The mass anthelmintic treatment reported as a risk factor of AR [4] was implemented in almost all participating herds. Targeted selective treatment that can reduce selection pressure for AR [30] was implemented in only one herd. A considerable proportion of farmers introduced goats from other herds without an appropriate protocol for the prevention of importation of diseases. AR can also be spread by imported animals carrying AR parasites [14,22]. Thus, examining new goats for AR before the introduction is critical [18]. These unsuitable management practices may have contributed to albendazole and ivermectin resistance in study populations.

Based on statistical analysis and data mining, frequent use of anthelmintic and new goat introduction might be responsible for levamisole resistance in study herds. More study populations with a variation of AR status are required to determine factors critical for the development of AR. A case-control study and a survey questionnaire based on AR status of herds would be suggested for further study to determine factors critical for the development of AR.

All farmers in the present study were informed of AR status in their herds by the research team, as recommended by Crook *et al*. [26]. Levamisole was suggested for anthelmintic treatment in this area. Adopting targeted selective treatment and appropriate use have been proposed to preserve the efficacy of this anthelmintic. Regular or periodic monitoring of the efficacy of anthelmintics would be helpful to manage AR. The FECR test used in the present study does not require complicated equipment and procedures, and thus, it can be implemented in the field by trained personnel. Further studies on alternative control of GIN including herbal anthelmintics and biological control should be conducted to find the most appropriate method to minimize the occurrence of AR against chemical anthelmintics in the area.

## Conclusion

Our study shows a high prevalence of AR, especially to albendazole and ivermectin, in goats in In Buri district. Levamisole was still effective; however, it should be used in a program to minimize selective pressure on GINs and the occurrence of resistance. Local veterinarians should provide information and promote an education campaign to establish sustainable GIN control. In addition, the efficacy of anthelmintics should be routinely monitored to ensure their effectiveness.

## Authors’ Contributions

NR and SP: Conceived and designed the study. NR and NT: Conducted experiment. NR and SP: Analyzed the data, drafted and revised the manuscript. All authors read and approved the final manuscript.
